# Symmetries and synchronization from whole-neural activity in *C. elegans* connectome: Integration of functional and structural networks

**Published:** 2024-09-04

**Authors:** Bryant Avila, Pedro Augusto, David Phillips, Tommaso Gili, Manuel Zimmer, Hernán A. Makse

**Affiliations:** 1 Levich Institute and Physics Department, City College of New York, New York, NY 10031, USA; 2 Department of Neuroscience and Developmental Biology, University of Vienna, Vienna Biocenter (VBC), Vienna, Austria; 3 Vienna Biocenter PhD Program, Doctoral School of the University of Vienna and Medical University of Vienna, Vienna, Austria; 4 Mechanical Engineering Department, University of New Mexico, Albuquerque, NM 87131, USA; 5 Networks Unit, IMT Scuola Alti Studi Lucca, Piazza San Francesco 15, 55100, Lucca, Italy; 6 Department of Radiology, Neuroradiology Service, Memorial Sloan Kettering Cancer Center, New York, NY 10065, USA; 7 CUNY Neuroscience, Graduate Center, City University of New York, New York, NY 10031, USA

## Abstract

Understanding the dynamical behavior of complex systems from their underlying network architectures is a long-standing question in complexity theory. Therefore, many metrics have been devised to extract network features like motifs, centrality, and modularity measures. It has previously been proposed that network symmetries are of particular importance since they are expected to underly the synchronization of a system’s units, which is ubiquitously observed in nervous system activity patterns. However, perfectly symmetrical structures are difficult to assess in noisy measurements of biological systems, like neuronal connectomes. Here, we devise a principled method to infer network symmetries from combined connectome and neuronal activity data. Using nervous system-wide population activity recordings of the *C.elegans* backward locomotor system, we infer structures in the connectome called fibration symmetries, which can explain which group of neurons synchronize their activity. Our analysis suggests functional building blocks in the animal’s motor periphery, providing new testable hypotheses on how descending interneuron circuits communicate with the motor periphery to control behavior. Our approach opens a new door to exploring the structure-function relations in other complex systems, like the nervous systems of larger animals.

## 1 Introduction

Complex systems depend on an intricate interplay between the dynamical properties of their building blocks and the network structures that link them together. For decades, researchers have endeavored to uncover how the topological attributes of a network could shed light on its dynamical behavior. While several methods have been proposed to elucidate this interplay,^1–4^ the role of the network’s symmetries has garnered considerable attention in recent years.^5–12^ Identifying and understanding such symmetries has potentially profound implications: network theory implies that certain symmetrical structures termed fibration symmetries in a graph permit synchronizations,^7,8,10,12^ a ubiquitously observed phenomenon in brain networks.^13^ Thus, identifying these symmetries might be crucial for decoding some of the structure-function relationships in a neuronal network.

Fibration symmetry refers to a specific type of symmetry where nodes can be grouped into equivalence classes of balanced colorings based on their input patterns.^10,12^ Nodes in the same class receives identical ’input trees’, which are hierarchical mappings of input connectivity patterns, are proposed to share the same dynamics, and thus can synchronize their behavior. These equivalent nodes are said to belong to the same ’fiber’ and are also ’balanced colorings’ of the graph.^8^ This is formalized through admissible ordinary differential equations (ODEs), which describe the evolution of the system’s state.^5^ Theory ensures that balanced colored nodes with isomorphic input trees following a set of admissible ODEs will evolve in synchrony^7,8,12,14,15^ forming a cluster of synchronization.^5,6^ This synchronization can be crucial in various biological systems ranging from gene regulatory networks to brain networks involved in language processing.^16,17^ Thus, identifying fibration symmetries provide a prediction in which subsets of nodes potentially exhibit synchronized dynamics.

However, there are technical and conceptual challenges to this theory. Biological networks like connectomes are inherently incomplete, and measuring them is limited by annotation errors, missing links, and noise, yet identifying fiber symmetries is sensitive to small variations in a connectivity matrix. More fundamentally, assuming mathematically perfect symmetries and equivalences of nodes in a biological network is unrealistic. Here, we propose that network symmetries represent constraints rather than perfect blueprints for their implementations in individual animals. We aim to address these challenges by studying the interplay of symmetry and synchronization in the locomotor system of the nematode *C.elegans*.

The nematode worm *C.elegans*^18–20^exhibits a small nervous system of just 302 neurons that develop from a stereotypic cell lineage into 118 anatomically and genetically defined cell classes, most of which are comprised of a bilaterally symmetrical pair of neurons (e.g., AVAL and AVAR).^21^ Its connectome has been fully reconstructed with a synaptic resolution,^21–23^ and it is tractable for large-scale single-cell resolution neuronal calcium imaging.^24,25^ Both, in combination, offer unique opportunities to study structure-function relationships in the nervous system.^4^ The worm connectome shares many features with connectivity data from larger nervous systems, e.g., rich-club architecture and modularity.^23,26–28^ Moreover, neuronal recordings in *C. elegans* revealed nervous system-wide neuronal activity dynamics that exhibit synchronization patterns among well-defined ensembles of neurons.^24,25,29^ These dynamics correspond to motor commands for a set of actions like forward-crawling, backward-crawling, or turning.^24,30^

These dynamics are generated in the absence of any known acute time-varying sensory stimuli^24,25,29^ and can be promoted by arousing conditions, such as environmental oxygen or blue light.^31,32^ Moreover, these dynamics can be observed in immobilized animals; even under such conditions, they extend to the motor periphery, incorporating motor neurons, under unconstrained conditions mediating movement execution.^29^ These features point toward intrinsic mechanisms that drive and maintain synchronous activity states. In accordance, our previous work indicated that rich club architecture and input similarities are crucial architectural features in the connectome that permits synchronized neuronal dynamics.^25^

In the present study, we focus on the chemical synaptic network of the backward-crawling locomotion circuitry of *C. elegans* to circumvent some of the technical and conceptual constraints posed by theory and computational limits. This sub-network of just 21 individual neurons includes three bilateral pairs of interneurons, AVA, AVE, and AVD, which are situated in the head of the animal and send descending chemical synapses to two classes of motor neurons, termed DA1–9 and VA1–12. These motorneurons form neuromuscular junctions with the dorsal and ventral body wall muscles, respectively, and are required to generate the backward crawling gait.^33–35^ All chemical synapses in this sub-network are cholinergic and, with few exceptions, neurons within the same class share similar morphology and gene expression patterns.^20,21,36^ Hence, assuming equivalence among nodes within the same class could be a reasonable simplification.

By leveraging advanced calcium imaging techniques, we first study neuronal dynamics, aiming to identify patterns of synchronization within its backward locomotion circuitry. We then use the observed synchronization dynamics to infer underlying symmetries in the connectome.

Given the potential variability in connectomes across individual worms, expecting the universal connectome model to predict the exact neural synchronization across neurons is unrealistic. This variability underscores the necessity of adopting tailored approaches to understand the dynamic behavior from the structure of the connectome. We approach this problem by reconstructing the underlying connectome of *C. elegans* guided by the synchronization dynamics obtained experimentally. This is done by ”repairing” the connectivity structure of a typical connectome^26^ by solving an appropriately modeled integer linear program to find the minimal number of repairs of the available connectome needed to ensure the observed synchronization dynamics. We aim for a fibration-symmetric connectome that reflects the observed synchronization. This method provides a means to idealize connectomes conceptually. According to inter-animal connectome differences reported experimentally in,^27,37^ the threshold for connectome modifications can be taken to not exceed approximately 50%. However, for our final repair solution we take the stringent threshold of 25% given by animal-to-animal differences found in.^26^

To ascertain the robustness and validity of our solutions, we engage in a rigorous testing procedure. We aim to determine the optimality of our solution by reshuffling the neuronal labels in the connectome and revisiting the repair process. We accept our method’s solution only if the post-relabeling solutions consistently underperform our primary findings (in over 95% of instances).

Our study bridges the gap between structural and functional neuronal data, leveraging the power of symmetries in graph theory to shed light on synchronization dynamics within *C. elegans* and the structure of the connectome underlying this synchronization.

## 2 Results

**Fig 1.**
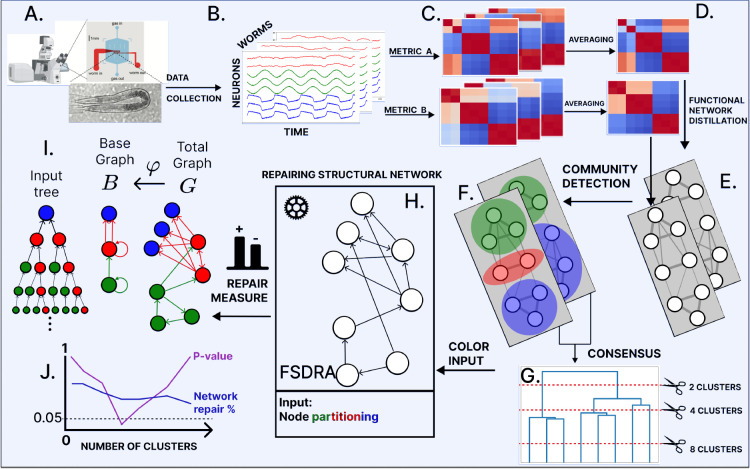
Pipeline for the structural network repair of the backward locomotion of *C. elegans* based on neural recordings. (A) Experimental setup. (B) Time series data showing the activity traces of multiple neurons. Traces are color-coded to indicate similar dynamics. (C) Matrices of synchronization are obtained from the traces. (D) The synchronicity matrices are averaged across worms, accounting for any missing activity traces of individual neurons. (E) The percolation procedure was applied to the averaged matrices to obtain the functional network. (F) Averaging the functional networks leads to a consensus matrix across different methods of synchrony measured. (G) A hierarchical partitioning is implemented to identify clusters of synchronicity. (H) MILP repairs the network according to cluster synchronization. (I) The solution to the MILP produces an ideal network guided by synchronization. This network can be ”collapsed” into its base. (J) p-value statistics to choose the optimal solution.

In this section, we delineate the pipeline employed throughout our study in a condensed fashion and details are elaborated in the Methods section 4. Figure 1 provides an outline of the method to reconstruct the connectome:

1. The process begins with the experimental setup to record neuron activity in Fig. 1A with a microfluidic device for immobilization of *C. elegans* enabling nervous system-wide Ca++ imaging with confocal fluorescence microscope setup.
2. Time series data for activity traces of multiple neurons are recorded simultaneously, as shown in Fig. 1B. This procedure is performed on multiple worms under similar conditions.
3. To obtain functional pairwise synchrony matrices, various metrics that capture synchrony are applied to the recorded time series data(Fig. 1C).
4. The synchrony matrices shown in Fig. 1D are averaged across worms to account for trial-to-trial and/or inter-individual variability.
5. A standard thresholding process is then applied to obtain the functional network (Fig. 1E).^38,39^ Starting from a disconnected graph, we add links between nodes in decreasing order of weight of the averaged synchrony matrix. This is done for each averaged matrix.
6. Finally, a consensus matrix is calculated^40^ across different methods of synchrony (Fig. 1F).
7. A hierarchical clustering algorithm is implemented to find a partition of synchrony clusters (Fig. 1G). Each neuron is assigned a color according to its cluster of synchrony. These colors are used as inputs to the repair algorithm in the next step.
8. We formulate and solve a mixed integer linear program (MILP) that finds the minimum number of edges to add or remove from the raw connectome (taken from^26^) to produce an ideal fibration-symmetric network that reproduces the coloring in the consensus partition obtained in the previous step (Fig. 1H).
9. The network produced by the solution to the MILP is a fibration symmetric connectome with cluster synchronization that reproduces the experimental data. This network can be collapsed into a smaller representation, the base graph, where nodes belonging to the same fiber have isomorphic input trees (Fig. 1I). The base graph suggests functional building blocks in the connectome.
10. For each consensus partition, a permutation p-value test is performed by permuting node labels and repairing the structural network 1,000 times. The partition with the lowest p-value is chosen as the optimal solution (Fig. 1J).

### 2.1 Neuronal activity recordings

To simultaneously record calcium activity from interneurons in the head and motor neurons along the entire ventral cord, we modify a recently reported whole nervous system imaging pipeline.^29^ Briefly, worms expressing the calcium probe NLS-GCaMP6f in a pan-neuronal fashion, together with NeuroPal multi-color cell identification labels,^41^ are immobilized in a microfluidic device that positions them in the field of view of a spinning disk confocal microscope. After fast volumetric GCaMP6f imaging, multi-color stacks are taken for cell class identification (see Methods section 4.1 for details).

### 2.2 Whole nervous system recording and characterization of motor neuron activity

**Fig 2.**
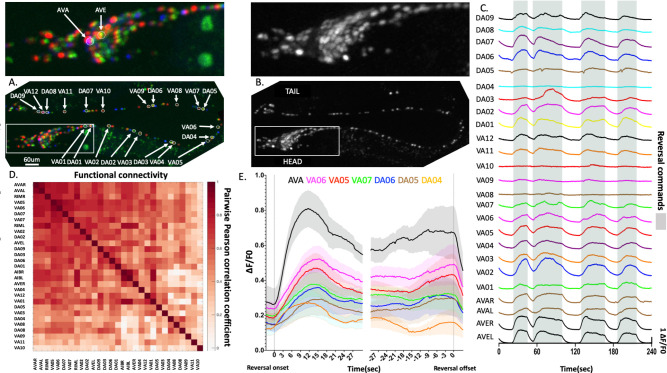
Whole nervous system recording reveals synchronous motor neuron activity. (A) NeuroPAL labeled worm with selected neuronal cell class identities indicated. (B) The same worm as in (A) showing NLS-GCaMP6f labeling. (C) Activity time series (DF/F0) of selected, grey shading indicates reversal command states defined by AVAL activity. (D) Pairwise Level of Synchronization (LoS) among selected neurons. Each matrix entry is the average of N=3–8 pairwise observations. (E) The triggered average (±SEM) to reversal command onset (left) and offset (right) are both defined by reference neuron AVAL. Three example neurons of each VA and DA motor neuron class are shown. Averages calculated across recordings represent the dynamics that we observe in our datasets; neurons are relatively aligned in time, but there are substantial differences regarding their amplitudes.

We generated eight datasets from different young adults well-fed worms. Imaging covered almost the entire body spanning from the head ganglia, the complete ventral cord, and the tail ganglia with single-cell resolution (Fig. 2A–B). Animals were recorded for 10 minutes at approximately three volumes per second. Figure 2C shows a multi-neuron time series with discernible calcium activity patterns of the neurons selected for this study. These include neurons AVAL/R and AVEL/R, which belong to the major descending interneurons conveying motor commands for backward crawling, as well as the downstream backward crawling motor neurons of the DA and VA classes. Consistent with previous studies,^24,25,29^ AVA and AVE activity exhibited discrete transitions in their activity patterns, characterized as low, rise, high, and fall states. We previously validated that these states correspond to behavioral states in freely crawling animals, i.e., low corresponds to forward crawling, rise and high corresponds to backward crawling, and fall corresponds to turning.^24^

Here, we observe that these features are mirrored in the motor periphery (Fig. 2C). The mean pairwise correlations among motorneurons and interneurons were high, indicating the strong coupling between them (Fig. 2D). During free backward crawling, animals generate a posterior-to-anterior traveling body wave of alternating dorsal-ventral body oscillations. We did not observe any obvious oscillations or activity patterns alternating between the DA and VA motorneurons, which innervate the dorsal and ventral body wall muscles, respectively. Neither did we observe any delay or sequential activation patterns between motor neurons that innervate more posterior to anterior muscles (which is indicated by the index number in their name, n=1 most anterior, n=12 most posterior). However, all motor neurons follow the rise and fall states of the AVA descending command interneuron (Fig. 2E). In conclusion, under our experimental immobilization conditions, A-class motorneurons do not generate gait-related patterns in their Ca++ activity profiles. However, they appear to respond to descending motor commands from their presynaptic interneurons reliably. This feature and the absence of movement, hence the lack of proprioceptive inputs, makes the present setup particularly suitable for studying neuronal dynamics dependent on internal neuronal circuit interactions (see also discussion).

### 2.3 Synchronization measures

Assessing the synchronization of two or more signals recorded simultaneously involves various methods tailored to the specific characteristics and similarities of interest. This results in a diverse array of techniques aiming to capture synchronization through different approaches.^42^ These techniques are generally classified into four primary groups based on their focus: time domain versus frequency domain and methods that account for directional dependencies versus those that do not (see Methods section 4.2 and Table 2).

Correlation is a robust measurement for coherence in neuronal data obtained via calcium imaging^24,43,44^ (Methods section 4.2). In addition to correlation, to quantify the amount of synchronicity between two signals, we implement the Level of Synchronicity (LoS) measure introduced in.^45,46^ This metric determines how closely two signals are to perfect synchronicity, meaning that two signals have the same value at the same time, forming a cluster of synchrony:

(1) $$V_{i}\left(t\right)=V_{j}\left(t\right)\;\;\forall i,j\in C_{k}.$$

Here, $V_{i}$ is the dynamic of a neuron, which, in the present case, is associated with the neuron’s calcium activity. $C_{k}$ is one of the non-overlapping sets of neurons partitioning the neural system into *Cluster of Synchronicity* (CS).^5^

To quantitatively capture this synchronicity, the LoS evaluates the synchronization of two signals over time by considering their instantaneous differences and scaling them through a parameter σ defined as:

(2) $$LoS_{ij}=\frac{1}{T}\sum_{t}^{T}\exp\left\{-\frac{{\left[V_{i}\left(t\right)\;-\;V_{j}\left(t\right)\right]}^{2}}{2\sigma^{2}}\right\},$$

where T is the total amount of time steps in which LoS is measured between the signals of neurons i and j. The parameter σ serves as a scale to define a benchmark for closeness between two points in time.

This parameter permits the user to deal with natural variations in biological systems. No two neurons are perfect copies of each other; therefore, if identical signals stimulate two similar neurons at rest, their outputs (membrane potential) will naturally vary in intensity and phase by small amounts. With this concept in mind, we implement multiple versions of the LoS, each with a different value for σ. When the value of σ is zero, each signal will only be synchronous with its exact copy; as the value of σ increments, it reaches the state that all signals are synchronous with all others. This indicates that an ideal value lies between zero and an upper limit (the largest difference between signals at some time t should suffice). After many trials, we settle for values ranging from 0.01 up to 0.20 to explore different levels of synchronicity. We proceed with two classes of synchronization metrics, correlations and LoS, each having variations through different parameters or relational measurements, making for a total of 44 metrics.

Matrices of synchronization and correlation are computed for each of the N=8 worms under study, capturing the synchronous activities observed within each individual as depicted in Fig. 1C. To obtain a representative overview of synchronicity across the entire set of worms, these individual matrices are averaged per type, i.e. for each value of σ (Fig. 1D). However, compiling these averaged matrices require care, particularly in instances where the activity of individual neurons are missing for specific worms. Therefore, we calculate the average by averaging over worms for a particular metric type and divide each element (neuron-pair functional measurement) in the summed matrix by the number of times they appear together across the whole cohort of worms.

### 2.4 Identifying cluster synchronization from the functional network

Each element in the averaged matrix contains information on a neuron pair synchronous relationships used to construct functional networks composed of all the neurons for the backward locomotion gait. We follow standard thresholding procedures^17,38,39^ to build the functional network. Through this method, we purge the functional data to only contain the strongest links leading to a functional network, as seen in Fig. 1E. Using this network, we identify neuron cliques that are synchronized via community and cluster detection algorithms.

A group of neurons that are more synchronous to each other than other neurons belonging to different groups is considered equivalent to a CS of neurons. We apply cluster and community detection algorithms to extract these functionally synchronous clusters of neurons from the functional network using two methods:

- Clique synchronization:^17^ It decides if a node belongs to a synchronous clique if the average value of the edges of the functional network inside the clique is bigger than any of its outside edges (see Methods section 4.3).
- Louvain community detection:^47^ It decides the number of clusters and the number of nodes in each cluster by optimizing the modularity function as given by Eq. (5) in the Methods section 4.3, which is solely cluster dependent.

**Fig 3.**
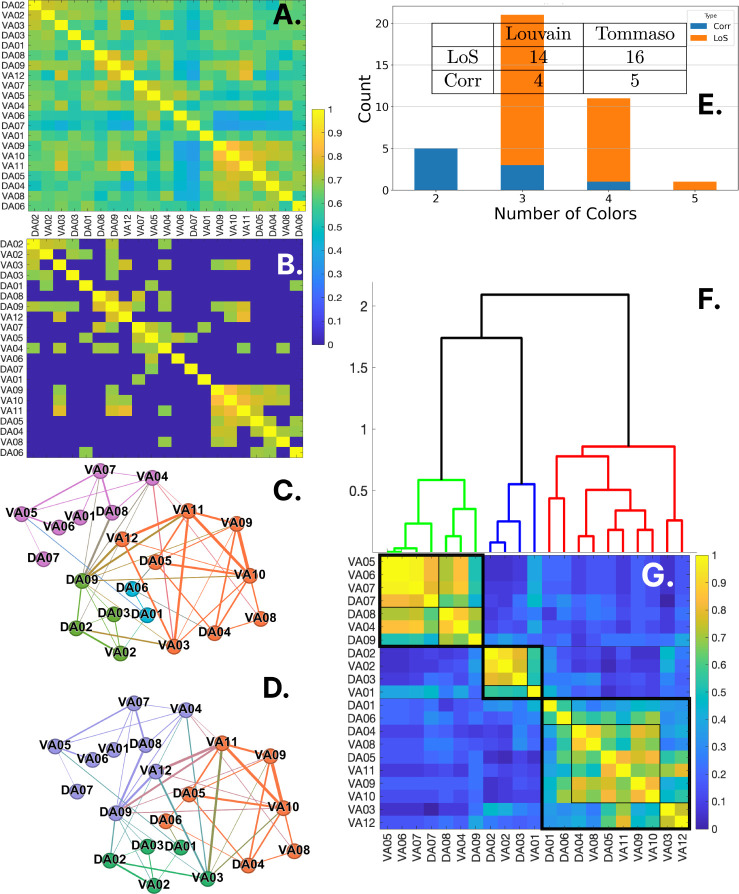
Results of the clustering analysis. (A) Average of N=8LoS matrices at σ=0.16. (B) Functional matrix thresholding removes elements smaller than the smallest and largest values for all neurons. (C) The functional network is converted into an indirect graph using the percolation thresholding method. Node colors show clustering using the Clique Synchronization method. Edge thickness relates to values in the LoS matrix. (D) Same as the previous functional network but with the Louvain method partitioning nodes. (E) Distribution of synchrony cluster numbers among unique partitionings. LoS measurements lead to partitionings with more clusters compared to correlation measurements. LoS peaks at partitionings with 3 clusters; correlations peak at 2 clusters. Given the neurons’ high monotonic and synchronous relationships, LoS measurement helps distinguish partitionings with more clusters via Ca2+ signal amplitude. Partitionings are applied to 39 LoS matrices and five correlation matrices using Distance, Pearson, Kendall, Spearman, and Covariance. LoS row matrices are obtained from unique *σ* values ranging from 0.01 to 0.20 in 0.005 steps. (F) Dendrogram of averaged co-occurrence matrices using Ward-metric hierarchical clustering. Three major groups of backward motor neurons are observed in green, blue, and red. (G) Averaged co-occurrence matrices show two levels of hierarchical clustering. Diagonal blocks with thick lines correspond to colored neuron groups from the dendrogram, while those with thin lines are sub-clusters within the three major clusters. These clusters correspond to a dendrogram slice at 1.00.

The result of applying these methods to a LoS correlation matrix is seen in Figs. 3A–B, which results in a functional network with the node partition as seen in Fig. 3C. The nodes with the same colors belong to the same cluster synchronization, as can be seen since they share thicker edges than edges between nodes of different colors.

The clusters obtained using the LoS measure at different parametric values produce various motor-neuron partitionings. We only keep those that are unique among the 39 results for the range of σ between 0.01 and 0.20. This is done for each of the two cluster/community detection methods. The number of unique partitionings for each method can be seen in the table within Fig. 3E.

### 2.5 Consensus synchronous clusters

We construct 44 metrics to obtain synchronization information: 39 LoS matrices and five types of correlation measures (Pearson, Spearman, and Kendall coefficient, distance correlation and covariance). We combine these metrics with the two functional clustering methods mentioned in the previous section 2.4 (clique synchronization and Louvain) to create 88 possible partitionings. Then, a consensus is created following the methods developed in^40^ to leverage the information obtained from all the partitionings. To achieve this, each partitioning, which consists of n clusters of synchronous neurons, is used to create a co-occurrence matrix, which is simply a matrix with n diagonal blocks, one for each synchronous group, with value 1, and with all other values outside these blocks set to zero (see Fig. 3D). These matrices are summed up and normalized. The resulting consensus matrix is depicted in Fig. 3G. Through this, the sets of neurons that appear in the same synchronous group more frequently than with other neurons appear with higher values. This consensus avoids putting each partitioning in competition with the other and instead compiles them into a globally agreed-upon result.^40^

The consensus matrix, called X (Fig. 3G), has optimal leaf ordering of its rows and columns. Such is produced by the hierarchical clustering *Ward* metric (also known as Ward’s minimum variance method), applied to the dissimilarity matrix 1–X (as seen in Fig. 3F). This metric aims to minimize the total within-cluster variance. The goal is to choose the successive clustering steps to minimize the increase in the total within-cluster variance. This metric is particularly effective for creating clusters that are compact and have a roughly similar number of elements,^48^ which was our main reason for selecting it.

From this consensus, we can obtain various partitionings depending on where the dendrogram is sliced. When the dendrogram is sliced at a value of 1.00 we obtain three major groups as observed in the thick block structure in Fig. 3G. Each block can be further divided into smaller diagonal blocks if we reduced the threshold. For instance, 7 clusters are observed for a cutoff at 0.55 represented by the smaller blocks in Fig. 3G. In the next sections, we will see that this partitioning is the optimal solution obtained by the reconstruction algorithm with the least amount of modifications to convert the baseline Varshney connectome into a fibration symmetric solution (7 clusters for a cutoff at 0.55 in Table 1).

All these partitions are the input to the mixed integer linear programming algorithm that we designed to reconstruct the connectome with a minimal number of modifications, as explained next.

#### 2.5.1 Balanced coloring partitions, fibrations and cluster synchronization

**Fig 4.**
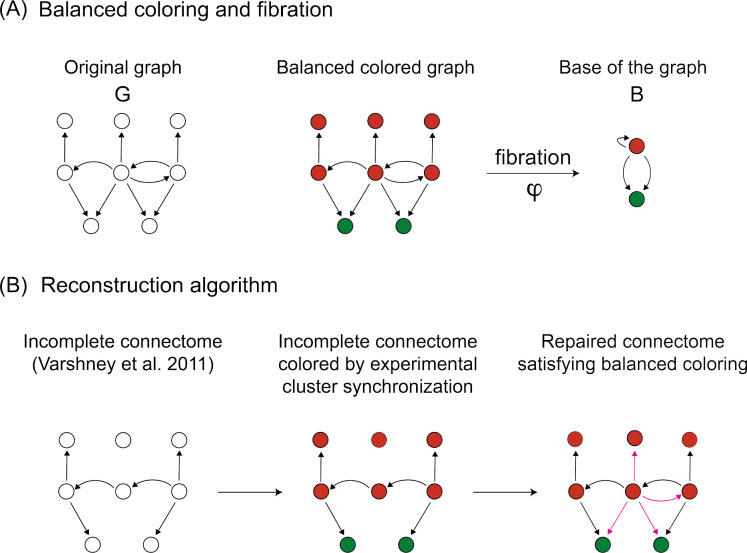
Fibration formalism and reconstruction algorithm. (A) Explanation of fibration symmetries and balanced colorings. The graph on the left has a (minimal) balanced coloring with 2 colors in the middle. For instance, each red node receives one red input and each green node receives two red inputs. The colored clusters represent clusters of synchrony when an admissible system of equations is superimposed on the graph. The graph *G* can be collapsed by the symmetry fibration to the minimal base as shown in the right panel, where the edges satisfy the lifting property. The colored clusters (also called fibers) are the building blocks of the network. A refinement algorithm to obtain minimal balanced coloring is provided in.^12^ (B) SymRep reconstruction algorithm. We start with an incomplete connectome (left) to which colors are assigned (middle) according to the cluster synchronization obtained experimentally. A MILP then adds (or removes) a minimal number of edges (in red) to construct an ideal network (right) that satisfies the experimental balanced coloring.

To develop the optimization algorithm to reconstruct the connectome to satisfy the synchronization found in the previous section, we need to introduce the concept of ’balanced coloring’ and the fibration of the graph. Figure 4A shows an example of a graph with a balanced coloring.

Let $𝒞=C_{1},\cdots ,C_{K}$ be a partition of the nodes of a network G=(V,E) with V vertices and E edges. We identify each cluster $C_{k}$ with a different color and K is the total number of colors. A ’balanced coloring’ is a coloring of the graph such that each node with color k in cluster $C_{k}$ is connected (by edges of the same type) to the same number of nodes with color j in cluster $C_{j},$ for 1≤k,j≤K. That is, nodes of the same color receive the same colors from their neighbors (Fig. 4A, center)

Translated into the terminology of dynamical systems, a system of differential equations for the state variables of each node $V_{i}(t)$ can be interpreted as a ’message passing’ process of passing colored messages through the edges of the graph. Since two nodes i and j with the same color received the same colors (messages) from their neighbors, we can think, intuitively, that they will synchronize their activity $V_{i}(t)=V_{i}(t)$, forming a synchronous cluster. This intuition is made mathematically rigorous^14^ through the theory of fibrations.^10,12^

Traditionally, a way to formalize the balanced coloring partitions is through the automorphisms of the graph forming its symmetry group.^5,6^ In this case, the orbits of the automorphisms of the graph are the balanced colors. However, not all balanced colorings are orbits. Many biological networks contain no automorphisms, yet, they display a non-trivial balanced coloring partition with many colors.^12,15^ This is exemplified in Fig. 4A. The graph has no automorphisms (except for the trivial identity), but has a balanced coloring that reduces the graph to a base with just two nodes. This balanced coloring is only captured by the fibration.

The graph fibration formalism introduced in^10^ is a more general formalism that captures all balanced coloring partitions of the graph. Graph fibrations are a particular case of fibrations between categories introduced previously by Grothendieck^49^ and are formal generalizations of graph automorphisms.

A graph fibration (shown on the right of Fig. 4A) is a morphism of the graph that collapses every cluster of synchronized balanced colors (called ’fibers’) into a single representative node in the base graph B while conserving the ’lifting property’ defined in the Methods section 4.4. This transformation leaves invariant the dynamics in the graph and captures the maximal symmetries of the network. It is then called a symmetry fibration.^12^ Here, we propose that fibers represent potential functional modules or building blocks in a network of neurons.

### 2.6 Fibration symmetry driven repair algorithm

**Fig 5.**
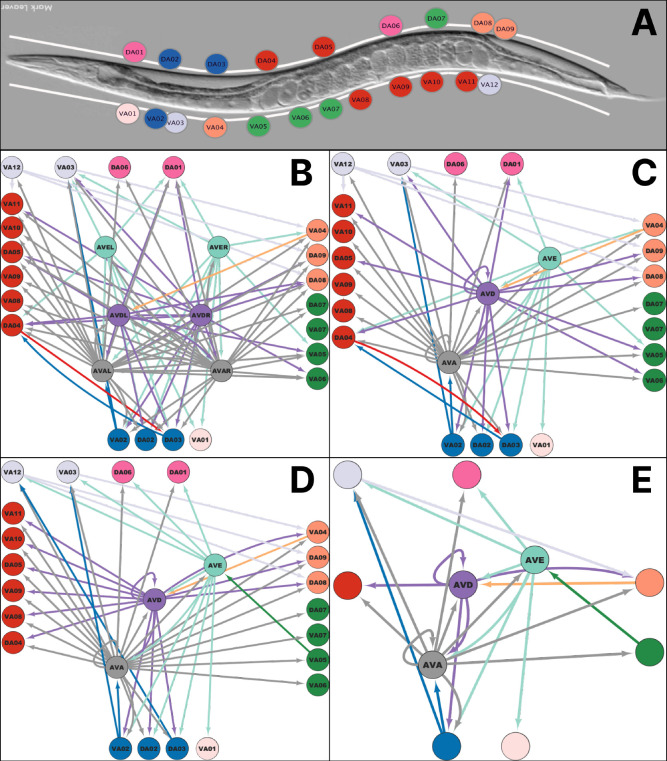
Reconstruction of the locomotion connectome. (A) Anterior-posterior distribution of motor neurons (DAn, VAn) involved in the backward locomotion in the *C.elegans*^51^ colored according to the most optimal consensus cluster synchronization method with cutoff 0.55 producing 7 colors. (B) Uncollapsed Varshney connectome from:^26^ Original Varshney chemical synaptic connectivity backward motor neurons and their three primary interneuron pairs AVAL/R, AVEL/R, and AVDL/R. (C) Collapsed Varshney connectome: Left-right collapsing of the interneurons in the original network in (B). This is done by joining left and right interneuron pairs into one unit and applying OR logic on their outgoing edges, given by the pair of adjacency matrix values for those sharing the same target neuron for each left and right interneuron pair taken as the source neurons. (D) Reconstructed network obtained by the MILP optimization algorithm, SymRep, applied to the network of (C) using the colors obtained from the synchronization analysis in Fig. 3G. This network is perfectly balanced colored: all neurons belonging to a specific colored cluster receive the same amount of colored input from other neurons. (E) The base graph representation of the network in (D).

The clusters of synchronous neurons found in section 2.5 are used to repair the raw chemical synapses connectome of the backward locomotion gait of *C.elegans* provided by Varshney *et al.*^26^ (plotted in Fig. 5B). We develop an optimization mixed linear integer program to modify this network by adding or removing the least amount of edges within the limits of an objective function^50^ to match the synchronization obtained experimentally. We call this algorithm the Symmetry-Driven Repair Algorithm or SymRep for short.

The goal is to obtain a network with balanced coloring partitioning as provided by one of the synchronization clusters from Fig. 3 (details appear in the Methods section 4.6). The most important feature of this algorithm in the context of the present paper is the objective function used to determine optimal solutions. Many combinations of adding and/or removing edges can satisfy turning the connectome into a network with color partitionings as provided by a functional clustering while respecting the constraints provided by Eq. (10) and Eq. (11). Of these many solutions, the one that minimizes the objective function below provides an optimal solution (Methods section 4.6):

(3) $$f_{\alpha ,\beta }\left(r,\;a\right)=\alpha \sum_{i,j\in E}r_{ij}+\beta \sum_{i,j\in E^{C}}a_{ij}$$

where E is the set of edges present in the original connectome and $E^{C}$ is the set of non-existing edges in the original connectome that are permissible to be added. The terms $r_{ij}$ and $a_{ij}$ are binary variables, where the first term, associated with E, takes on a value of 1 if an edge has been removed. The second term, associated with $E^{C}$, takes on a value of 1 if an edge has been added. If necessary, one can control the SymRep algorithm to prohibit removing (adding) connections between physically impossible neurons, which studies show are always connected (disconnected). This can be done by simply not including them in E or $E^{C}$. The parameters α and β are penalty weight constants for each of these variables, respectively. The relative weight between these parameters determines if the SymRep prefers to repair a network through the addition of edges rather than removal. In our study, we explore the solutions found by varying the relative differences between α and β. We keep the value of α at one and increment the value of β in steps of one, starting from one and culminating at 10.

We work with the raw chemical synapses connectome of *C.elegans* for the backward locomotion gait provided in.^26^ This is composed of the connectivity between motorneurons (VAs and DAs) and 3 main interneuron pairs (AVAL/R, AVEL/R, AVDL/R). We specifically use two versions of this connectome. One is that reported by Varshney *et al.*^26^ shown in Fig. 5B. We call it the Uncollapsed Varshney connectome. The second, shown in Fig. 5C, is a collapsed version of this network in which we assume that motorneurons can not distinguish between signals received from the left or right version of an interneuron pair (i.e., AVAL or AVAR). This is done by substituting an interneuron pair for one node (i.e., AVAL and AVAR are collapsed into AVA) and substituting the two edges a neuron may receive from an interneuron pair for one edge with a weight equal to 1 if both edges are present, one if only one edge is present, and 0 if not present. This version is called the Collapsed Varshney connectome. As a final note, we assume each interneuron pair belongs to its unique synchronization cluster.

### 2.7 Synchronization-driven reconstruction of the connectome

With the 39 unique clusterings found in the previous section, the 19 different combinations of weight penalties and the two different raw connectomes (original and collapsed versions) used, we run SymRep to produce 1,482 solutions. All of these solutions satisfy the condition of balanced coloring. Of the 741 solutions for the collapsed Varshney connectome, 730 (98.52%) satisfy the stricter condition of minimal balanced coloring, and 737 (99.46%) fulfill this condition for the not collapsed version. Of these, we present the most optimal solution for each type of cluster or community detection algorithm in combination with the functional measurement group (LoS and correlations).

We decide on an optimal solution based on the number of modifications it enacts on the raw connectome with the condition that this number is the lowest, and at least below 50% of natural variability found from animal to animal^27,37^ to accept the solution. We pick those that modify the connectome by the least amount of addition and removal of edges. We consider two cases to measure the number of modifications. The first one is where adding or removing has a cost of 1, where the final sum of modifications is divided by the total number of edges in the raw connectome. The second case is in which the severity of removing an edge is tied to the weight of the edge in the collapsed connectome before binarization while adding an edge only has a cost of one. In the latter, the total sum of modifications (additions + removals) is divided by the total number of edges in the raw connectome. Metadata about the solutions can be observed in Table 1 with significant p-values for each method.

### 2.8 Statistical significance

**Fig 6.**
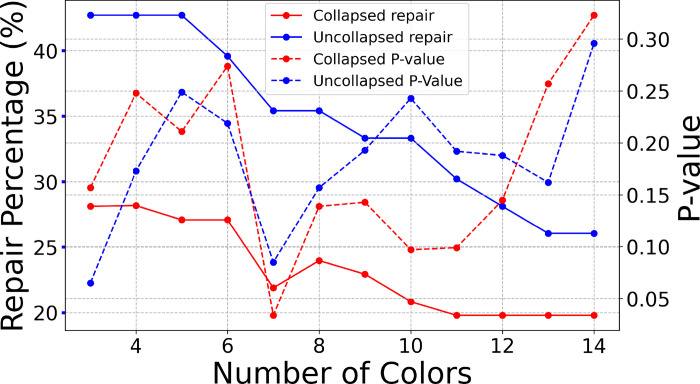
Statistical analysis. The plot visualizes the percentage of edge modifications required to achieve fibration symmetry in networks as a function of node consensus partitioning into *N* clusters, represented along the x-axis. Some of these can be seen in Figs. 3H–I. The graph compares repairs done on the ’Collapsed’ and ’Uncollapsed’ backward gait network of the *C.elegans* with the percentages needed for different numbers of colors (clusters). Additionally, we show the statistical significance (P-values) of these solutions against 1,000 permutations with similar cluster distributions but shuffled node labels. The dual y-axis format highlights the repair percentages (continuous lines) and their P-values (dashed lines), providing a comprehensive view of the network modifications required to achieve fiber symmetry in this network. The lowest p-value (and below the standard 0.05 p-value cutoff for acceptance) with repairs as low as 22% are found for 7 clusters at consensus clustering cutoff of 0.55.

To test the significance of these functional partitionings and eventually choose the most significant partition, we proceed to use a permutation test.^52^ Specifically, for each of the partitionings considered in Figure 6 we randomly permute the labels (names) of the neurons, keeping the number of clusters and number of nodes distributed among these the same. This leads to a shuffling of synchronous groups keeping their size (amount of neurons in each) the same. We produce slightly over 1,000 permuted versions of each of these partitionings and inspect how many lead to networks with these partitionings as fibers with equal or less number of edge modifications. This quantity is then divided by 1,000 or by the number of networks that lead to a minimally balanced coloring solution with an equal number of fibers as in the partitionings (~1,000).

### 2.9 The optimal solution

The consensus matrix allows the detection of fibers at different scales (number of clusters) through the information contained in its hierarchical clustering, as seen in Fig. 3F. These fibers are used for repairing the Varshney chemical synaptic backward gait produces some outstanding results among the collapsed and non-collapsed connectome. We highlight the results for the collapsed connectome at the bottom portion of Figure 6.

When it comes to the statistical significance of clusters obtained through a system of ever-increasing node partitionings to repair a network, as in our case, there is some expected behavior. For instance, the trivial partitioning of N neurons into N non-overlapping clusters will give a p-value of 1 as all permutations of a trivial partitioning will produce the same partitioning, resulting in all of them modifying the network by the same amount. The same can be said for a trivial partitioning with 1 cluster as all permutations result in the same partitioning. Therefore, as the number of clusters change between 1 to N, the p-value of statistical significance is granted to produce a (global) minimum p-value between 1 and N clusters.^52^ For a low-noise system in which node dynamics are faithful to the network structure with 1≤M≤N fiber partitionings, it is suggested that as the number of non-overlapping clusters is reduced from N, the p-value is expected to improve (be below 1), with the same expected p-value as the number of clusters is increased above one where the minimum p-value should be located at M clusters. Deviations from this depends on how far removed the network under repair is from the original fiber symmetric version producer of the node mentioned above dynamics. These predictions in the behavior of p-value are observed in the repairing of the backward locomotion network in Fig. 6. As the number of clusters approaches the trivial N-partitioning the amount of repairs needed decreases because the network we are trying to repair is indeed originally trivially colored.

Applying all these considerations, we find that the most optimal partition and reconstructed connectome is obtained for 7 clusters at consensus clustering cutoff of 0.55 repaired from the collapsed Varshney connectome. As shown in Fig. 6 and Table 1 this solution has the lowest p-value=0.033, being the only one of the consensus solutions below the standard 0.05 p-value cutoff for statistical acceptance. Furthermore, the repair percentage is 21.88%, below the acceptance limit of 25% variability from animal to animal. Therefore, we choose this connectome, shown in Fig. 5A, as the one representing the data in the closest possible way.

## 3 Discussion

Understanding how the structure of the connectome influences the function of the network is a long-standing problem in system neuroscience.^1^ However, addressing this question is challenging due to low sample number in available connectome datasets and potential variability across individuals. We have addressed the problem of incomplete/missing data in biological networks by first developing a consensus method to obtain average clusters of neuron synchrony across worms from whole-body calcium recording of neural activity. This information drives a reconstruction optimization algorithm of the connectome consistent with the observed synchronization. When the modifications to the connectome are below the experimentally found variation from animal to animal and offer a minimal p-value significance, the obtained connectome models can be thought of as idealized networks consistent with the experimentally measured dynamics of the neuronal circuits. Such a network bridges the gap between structure and synchronization and can be used to further assess the functionality of the connectome via perturbations to its structure.

Our research has substantiated the pivotal role of fibration symmetries in the connectome structure in orchestrating neuronal synchronization in *C. elegans*. The refined understanding that structural connectomics underpins significant aspects of neural functionality furthers our grasp on the physical bases of neural synchronization. This synchronization is crucial, as it forms the foundation for coordinated motor outputs and behavioral responses in organisms. The use of advanced calcium imaging and graph theory to correlate these structural motifs with dynamic patterns of neural activity offers a compelling model for predicting neuronal behavior based on underlying anatomical data.

The methodological innovations in our study, particularly the use of integer linear programming (SymRep) to adjust connectomics data based on functional imaging, demonstrate a novel computational approach to neuroscientific research. This technique refines existing neural network models and provides a quantifiable method for aligning theoretical predictions with empirical observations.

Previous research in *C. elegans* has suggested local oscillators at the motor neuron level for rhythm generation in the VNC for reverse locomotion;^35^ these patterns have been observed when motor neurons were experimentally deprived of their descending interneuron inputs. Our experimental conditions, Ca++ imaging in immobilized worms with interneurons AVA and AVE generating descending motor commands, did not reveal any oscillations or activation patterns consistent with a rhythmic backward gait pattern i.e., alternations between VA- and DA-motorneurons, or a traveling wave from the posterior to anterior position. Instead, we observed that A-motor neurons faithfully mirror the descending motor command activity in sync. It was shown that *C. elegans* can adapt their gait flexibly to the physical properties of the environment via proprioception, as shown for forward locomotion,^53^ and that A-motorneurons have indeed proprioceptive properties themselves.^54^ We suggest that proprioception might be an essential driver to unleash the oscillatory or rhythmic properties observed in ref.^35^ In our study, the lack of proprioceptive inputs and absence of complex internal motorneuron dynamics was an advantage, since it enabled us to study the activity of all A-motorneurons solely in response to their synaptic partners, providing a filter on activity that should be mostly explainable by the connectome architecture alone.

Connectomes between adult worms are remarkably variable, in particular for connections between neurons with few synaptic contacts^27^ as well as in the posterior ventral cord (VNC).^37^ Extrapolating from these data, connections between descending interneurons and motor neurons in the entire VNC might vary by up to 50% i.e., about half of the connections found might not be reproducible across individuals. Our connectome repair algorithm provides a family of statistically significant solutions that require connection additions/removals well below this range (Table 1, Fig. 6). All of these solutions provide circuit models with fiber symmetrical properties consistent with synchronization patterns found in A-motorneurons. It is unlikely, that individual animals indeed perfectly match these idealized symmetric solutions, thus neuronal networks must be endowed with additional properties that ensure stable synchronous dynamics. However, here we propose that fibration symmetries provide constraints, from which individuals should not deviate too much to ensure the robustness of synchronous dynamics. Here we show that symetrization procedures can, however, be useful, for identifying potentially functional circuit modules in the resulting base graph.

Our analyses led to a base graph of the backward locomotion circuit that suggests functional modules characteristic of differential innervation by the descending interneurons AVA, AVE, and AVD (Fig. 5E), which intriguingly appear as a crude topographic map (Fig. 5A). Our analyses thereby suggest that these modules could operate as functional units in deferentially transmitting the motor commands to different body parts, perhaps in a manner supporting effective backward locomotion. AVA, AVE, and AVD differ to some degree in their connectivity to other sensory circuits;^21^ we thus speculate that the modules might enable differential control over how to execute the reversal motor command. Note, that AVD neurons were not active in our recordings. Future studies that activate also AVD, and selective inhibition or interference with AVA, AVE, and AVD will test the relevance of our findings for the control of A-motorneuron activity and backward crawling behavior.

The present study focused on a smaller neuronal circuit where some of the assumptions that our theory includes might be reasonable simplifications. Anatomically and molecularly, left and right members of interneuron classes AVA, AVE and AVD are nearly indistinguishable,^20^ justifying bilaterally collapsing the connectome (Fig. 6 and Table 1). Moreover, most chemical synapses in the circuit are excitatory and cholinergic,^36^ and VA and DA motorneurons have similar molecular footprints,^20^ justifying the simplification of node and link equivalency. Future work on larger circuits in the worm and other model organisms should develop our theoretical approach further to realistically allow for more diversity in neuronal and synaptic properties.

Indeed, the implications of our findings are not restricted to *C.elegans*. The principles of neuronal synchronization, facilitated by connectomic structures observed in this study, likely have analogs across bilaterian species, including humans.^17^ Investigating these principles in more complex nervous systems could reveal new insights into how brain-wide synchronization patterns contribute to complex behaviors and cognitive functions. Furthermore, exploring the impact of structural variations on the synchronization in disease models could open new therapeutic avenues, particularly for neurological disorders where dysregulation of synchronized activity is evident.

## 4 Methods

In this section, we first explain the experimental setup and the methods to extract the functional synchronization clusters of neurons obtained using various combinations of functional measurements and different methods of extracting clusters and communities. Following this, we introduce the graph fibration formalism to describe balanced coloring partitions of graphs and cluster synchronization. We conclude with the repair algorithm for optimal fibration symmetric network solutions (SymRep) applied to the synchronization partitionings.

### 4.1 Neuronal calcium imaging

Whole-nervous system Ca2+ imaging experiments were performed on transgenic young adult *C.elegans* hermaphrodites (age was determined by the number of a maximum of 5 eggs) expressing genetically-encoded calcium indicator NLS-GCaMP6f in a pan-neuronal fashion and localized to the cell nuclei^25,29^ together with NeuroPal cell class identification labels^41^ (ZIM2001: *otIs669; MzmIs52 ; lite-1 (ce314)*)

#### Mounting and microfluidic setup:

Animals were imaged in two-layer PDMS microfluidic devices to control the oxygen environment and with curved channels^25^ to immobilize and laterally align animals, enabling reliable positioning of worms across recordings and fitting them into the field of view of the imaging system. Such a layout allowed us to cover the head ganglia, ventral cord, and tail ganglia of the animals. The worm channel of the microfluidic device was connected to a syringe that contains NGM buffer with 1 mM tetramisole to paralyze worms. All components were connected using Tygon tubing (0.02 in ID, 0.06 in OD; Norton) using 23G Luer-stub adapters (Intramedic). Constant gas flow of 21% O2 and 79% N2 (50ml/min) was delivered using a gas mixer connected to mass flow controllers (Vögtling Instruments) using lab custom scripts in Micromanager. Adult worms were picked on food-free NGM agar plates in a drop of NGM with 1mM tetramisole and aspirated into the worm channel. Animals were first habituated for 10min and afterward imaged at 21% O2 for 10 min.

#### Microscope setup:

High-resolution data of neuronal activity in the head and tail ganglia were acquired with an inverted spinning disk confocal microscope (Zeiss Axio Observer.Z1 with attached Yokogawa CSU-X1) using a sCMOS camera (pco.edge 4.2 with Camera Link HS connection) and a 40× 1.2 LD LCI Plan- Apochromat water-immersion objective (Zeiss). Moreover, we used 0.5x demagnification relay optics at the port of the spinning disk unit to increase intensity/pixel and the total area that can be imaged on the camera sensor both by the factor of 2, allowing imaging of a larger field of view encompassing the full worm nervous system. We use a custom-made GUI in Micromanager to control the different elements of the microscope. Exposure time was 20 ms, with 2*μ*m steps between Z-planes operated by a Piezo stage (P-736 PInano, Physik Instrumente GmbH); the total plane number of z-planes varied between 16–20, leading to a volume acquisition rate of up to 3 Hz.

#### Neural trace extraction:

As described in detail in our previous work in Kato *et al.*,^24^ neuronal activity traces were obtained by tracking the intensity maxima in each volume over time and calculating the single-cell fluorescence intensities. F0 was calculated for every neuron as the mean fluorescence intensity across each trial. After background subtraction, DF/ F0 was calculated for each neuron, following bleach correction by linear detrending and exponential fitting. See reference^24^ for details.

#### Neuronal identification:

Identification of each neuron was done based on a neuron dictionary termed NeuroPAL^41^ (Fig. 2A). In each recording, we aim to detect at least 25 crucial neurons for our analysis: two reversal interneurons AVA and AVE, and all the reversal motor neurons DA01-DA09 and VA01-VA12. Some neurons in the head and tail tip were lost in individual recordings, depending on animal size, when it exceeded the imaging region. Other reasons for missing neurons might be a failure in segmentation and tracking due to low signal/noise ratios based on low expression levels and/or low calcium levels. Neuronal traces were curated after each recording, and obviously erroneous traces were removed. Average correlation matrices were generated by calculating the mean pairwise correlations between the activity time series of identified active neurons (up to n=8, depending on the number of pairwise observations, but at least n=3). The identified active neuron numbers are as follows: 72 for recording 1, 76 for recording 2, 98 for recording 3, 79 for recording 4 and 5, 69 for recording 6, 63 for recording 7, and 62 for recording 8

#### NeuroPAL labeling:

The identities of neurons were determined via NeuroPAL using the following procedure. We obtained image stacks from each recorded animal after GCaMP6f imaging. For each plane, we acquired spectrally isolated images sequentially of CyOFP1, mNeptune2.5, and mTagBFP2. We excited CyOFP1 using the 488nm laser at 5% intensity with a 585/40 bandpass emission filter. Afterward, mNeptune2.5 and TagRFP-T were recorded using a 561nm laser at 20% intensity (percentage of max) with a 655LP filter and a 570LP filter, respectively. mTagBFP2 was isolated using a 405nm laser at 30% intensities with a 447/60 bandpass filter. These imaging conditions allow for the color-specific labeling of *C. elegans* neurons. Neuronal identities were manually annotated according to the NeuroPal guidelines.^41^

### 4.2 The zoo of synchronization measures

Determining the synchronization between two or more simultaneously recorded signals is a task with multiple approaches depending on the characteristics that one is interested in measuring and determining their level of similarity. Naturally, all this leads to a zoo of methods that try to capture the synchronization between signals through different features.^42^ These methods tend to be broken into four main camps of measurement, which are the intersection of the time domain vs the frequency domain and those that consider directional dependencies and that do not; some of these examples can be observed in Table 2.

Depending on the data and the aspects of the signals one is interested in, one may use a few examples in Table 2 and even other methods not mentioned in the table. Selecting the appropriate metrics can sometimes be a difficult problem, and one must compare these against some reference, producing competition between the selected metrics. Below, we indicate which metrics we have decided to use. Further, down in the paper, it is shown that it is best not to put these metrics in competition one against the other to find the best metric that captures synchronization. Instead, a better approach is to use the information that these provide in a consensus manner to decipher the synchronization in our system.^40^

### 4.3 Clique synchronization and Louvain method

From section 2.4, the Clique Synchronization method developed in^17^ accepts a clique if all nodes composing a clique satisfy the conditions outlined in section 2.4. We define a cluster of N neurons to be synchronous as a fully connected clique consisting of N nodes that meet the conditions:

(4) $$\sum_{i<j}^{1,N}\sigma \left(x_{i}\left(t\right),x_{j}\left(t\right)\right)\geq \frac{N\left(N\;-\;1\right)}{2}\sigma \left(x_{k}\left(t\right),x_{k^{\prime }}\left(t\right)\right)\;\;\;\;\;\;\forall k=1,\ldots ,N\;\mathrm{and}\;\;k^{\prime }\in {ℳ}_{k},$$

where ${ℳ}_{k}$ denotes the set of nearest neighbors of node k (for k=1,…,N) that are not part of the clique in question. Here, $\sigma \left(x_{i}(t),x_{j}(t)\right)$ represents the value for synchronization, measured by the LoS metric or any correlation metric used in this paper.

The Louvain method^47^ is a popular algorithm for module and community detection. At every execution, it assigns every neuron to its community, as per the method used here, it proceeds to randomly group neurons into bigger clusters, only accepting mergers if the modularity value given by Eq. (5) increases and halting once this equation can no longer increase in value.^47^ Due to the stochastic nature of this process, each execution may lead to a slightly different result. Due to this, the Louvain method is executed 1,000 times for a given network retaining the partitioning with the highest measured modularity. The modularity measure is defined as:

(5) $$Q=\frac{1}{2m}\sum_{i,j}[A_{ij}-\frac{k_{i}k_{j}}{2m}]\delta \left(c_{i},c_{j}\right),$$

where $A_{ij}$ is the adjacency matrix of the network, m is the number of edges in the network, $k_{i}$ is the number of in-degree edges attached to node i and $c_{i}$ are numeric labeled given to each synchronization cluster.^47^

### 4.4 Fibration symmetry of graphs and cluster synchronization

In the study of complex networks, understanding the interplay between structure and dynamics play a crucial role. Specifically, the concept of synchronization, where nodes in a network adjust their behavior in accordance with each other is fundamental in various disciplines, including physics, biology, and engineering. This section delves into the intricate relationship between graph topology, characterized by fibration symmetry, and the emergence of synchronization through the lens of admissible ordinary differential equations (ODEs).^7,8,12^

A graph $G=\left(V_{G},E_{G}\right)$ consists of a set of vertices $V_{G}$ and a set of edges $E_{G}$ where each edge connects a pair of nodes u to v represented as $(u,v)\equiv e_{G}^{u\to v}$. The set of edges from node u to node v is written as $E_{G}(u,v)$, that is, the set of edges $e\in E_{G}$ that have as a source node u brought by the function s(e)=u and have as a target node v brought by the function t(e)=v. The set of edges in graph G that have as a target the node v is denoted by $E_{G}(-,v)$.^55^

A graph G can be mapped into another graph W through the structure preserving process of a morphism Ψ:G⟶W using a pair of functions $\Psi_{V}\;:V_{G}⟶V_{W}$ and $\Psi_{E}\;:E_{G}⟶E_{W}$. These functions map vertices to vertices and edges to edges, respectively, from graph G to W, while being commutative with the source and target mapping functions. In mathematical terms, these functions must obey: ^10^

(6) $$s_{W}\circ \Psi_{E}=\Psi_{V}\circ s_{G},$$

and

(7) $$t_{W}\circ \Psi_{E}=\Psi_{V}\circ t_{G}.$$

If the mapping functions $\Psi_{V}$ and $\Psi_{E}$ are surjective, that is to say, they map multiple elements (vertices or edges) in their domain (graph G) to one representative element in their range (graph W), then morphism Ψ is called an epimorphism.

A (surjective) graph fibration is a particular type of epimorphic morphism that maps elements that belong to the same category into one representative element while preserving the lifting property which guarantee dynamical invariance. The original definition of fibration was defined between categories by Grothendieck and others. ^49^ Boldi and Vigna ^10^ worked out the definition of fibrations between graphs, which is the main theoretical framework of the present work. Morone *et al*.^12^ then showed the application of fibration to biological networks to understand their building blocks, ^15^ and synchronization. ^16,46^

A **graph fibration** is a morphism from graph G to the base B:

(8) φ:G⟶B

that satisfy the lifting property.^10^ This means that for every edge $e=(u,\;v)\in E_{G}$, there is an edge $e^{\prime }=(\varphi (u),\;\varphi (v))\in E_{B}$, and for every vertex $v\in V_{G}$ there exists a unique edge $e^{v}\in E_{G}(-,\;v)$ satisfying $(w,\;\varphi (v))\in E_{B}$.

The importance of the lifting property is that the dynamics between the graphs G and B is preserved. This means that if we add a set of admissible ODEs to the graph with dynamical variables $x_{i}(t)$, then the dynamical evolution of the system in graph G is the same as the dynamical evolution of the graph B.


(9) $$Dyn\left(G\right)=Dyn\left(B\right).$$

This property defines the clusters of synchrony as follows.

When a function φ:G→B acts as a fibration, we refer to G as the total graph and B as the base graph of this mapping function. In this context, G is said to be fibred over B. The set of vertices in G that φ sends to a specific vertex x in B is called the fiber over x, denoted by $\varphi^{-1}(x)$. Fibers are also balanced color clusters.

Vertices belonging to the same fiber have isomorphic input trees as defined in.^12^ The input tree for a node v, denoted T(v), is a rooted tree centered at node v that captures all the paths in the graph leading to v. The first layer of the tree is the node’s in-neighborhood, called its input set. Each subsequent layer is then iteratively defined as the input set’s input set. A visual example of an input tree can be seen in Fig. 1I, where all blue nodes in the total space graph have the same input tree structure.

In terms of dynamics, we can think of a message passing process where the information travels through the input tree and arrives at the rooted node v. If another node u has an isomorphic input tree with v, meaning that T(v)~T(u), then these two nodes receive the same messages through the network, although from different pathways. Therefore u and v synchronize their dynamics $x_{v}(t)=x_{u}(t)$. This statement has been put in rigorous mathematical terms by DeVille and Lerman.^14^

A fiber is called trivial if it contains exactly one vertex, that is if $\left|\varphi^{-1}(x)\right|=1$ and called nontrivial if $\left|\varphi^{-1}(x)\right|>1$. Throughout this paper, the function |⋅| denotes the number of elements in the mathematical structure it encloses unless specified otherwise.

Vertices belonging to the same fiber, say u and v, have an equivalence relation ≃ called an in-isomorphism, which can be seen as a particular case of a discrete homeomorphism,^10^ where a discrete bijective function (one-to-one association of elements in two domains) ψ:G(-,u)→G(-,v) is applied to the discrete topological space of graphs.

Additionally, the **in**- in in-isomorphism is the added condition for the bijective function to obey s(e)≃s(ψ(e)) for all e∈G(-,u). In short, in-isomorphism holds the notion that the vertices in a graph G can be converted into one another while conversing the adjacency connectivity from the same (or equivalent) source vertices.

A fiber in a graph G is associated with the cluster containing the vertices of the fiber $u,v,w,\ldots \in C_{i}$ where $n_{i}$ represents the number of vertices in cluster $C_{i}$ with its vertices noted as $\cup_{l=1}^{n_{i}}v_{l}^{i}=C_{i}$. The union of these clusters contains the entirety of vertices $C_{1}\cup C_{2},\cup \ldots C_{K}\in V_{G}$ where K is the number of fibers in graph G. The overlap of different clusters is always empty $C_{i}\cap C_{j}=\emptyset$ for $i\neq j\;\forall i,\;j\in K_{G}$ where $K_{G}=\{x\in Z|1\leq x\leq K\}$ such that $\sum_{c=1}^{K}\;n_{c}=\left|V_{G}\right|$.

These fibers are the equitable partition or colored-balanced partitioning of the graph as the number of in-degree edges of the in-isomorphic vertices in a cluster $C_{i}$ receives from another cluster $C_{j}$ only depends on the choice of the clusters. Thus, both fibers and balanced colorings describe, in two different ways, the same synchronous dynamics of clusters of nodes.

We can represent this as:

(10) $$C_{i}↢C_{j}\equiv \{\left|\cup_{l=1}^{n_{j}}E_{G}(v_{l}^{j},v^{i})|=|\cup_{l=1}^{n_{j}}E_{G}(v_{l}^{j},v^{k})\right|\}\;\forall v^{i},v^{k}\in C_{i}$$

where the number of edges |⋅| every vertex in $C_{i}$ receives from a cluster $C_{j}$ must the same.^56^

The particular type of fibration we work with in this paper is the *minimal* fibration, which leads to a minimal equitable (colored balanced) graph partitioning. The word minimal enforces the value K representing the number of colored balanced partitionings of a graph to be the lowest it can be. This leads to the situation in which no two clusters receive the same in-degree edges relative to a third cluster, which leads to the constraint

(11) $$\sum_{k=1}^{K}\left|\left(C_{i}↢C_{k}\right)-\left(C_{j}↢C_{k}\right)\right|>0\;\;\;\;\;\forall i,j\in K_{G}.$$

The surjective minimal graph fibration is called a symmetry fibration^12^ since it collapses the graph into its minimal base capturing the minimal number of fibers (or balanced colors) of the graph, and thus collecting the maximal symmetries of the graph.

An implementation of the algorithm to find minimal balanced colorings in a graph in the form of an R package is available at https://github.com/makselab/fibrationSymmetries and https://osf.io/z793h/.

### 4.5 Fiber symmetries constrain admissible ODEs into synchronization

The fibration symmetry of a graph imposes a structural constraint that can facilitate synchronization. Specifically, if the graph has a fibration symmetry, the nodes in each fiber can be expected to synchronize with each other^14^ due to the consistent interaction patterns enforced by symmetry. This can be understood through the synchronization of a system of ordinarily differential equations (ODEs) ’admissible’ to a graph.^8,46,57^

Admissible means the structure of a graph G imposes the coupling terms between the $\left|V_{G}\right|$ number of ODEs, where each equation characterizes the state of a vertex in the graph. Consider a dynamical system on a graph where each vertex $v_{i}$ has a state $x_{i}$ that evolves according to an ODE:

(12) $$\frac{dx_{i}}{dt}=F\left(x_{i},t\right)+\sum_{j\in \partial_{i}}A_{ij}H\left(x_{i},x_{j},t\right).$$

Here, F represents the intrinsic dynamics of each vertex, H embodies the interaction between vertices, and $A_{ij}$ are the elements of the adjacency matrix A of the graph, where $A_{ij}=1$ indicates the presence of an edge and $A_{ij}=0$ indicates the absence of an edge from vertex j to i. $\partial_{i}$ denotes the set of neighbors of vertex i. As an example, the Kuramoto model, taken along with its master stability function can be used to study synchronization in a network.^5,58^

Cluster synchronization as defined by Pecora *et al*.^5^ in this context refers to the situation in which the dynamical states of a group of vertices for a given fiber converge to a common trajectory, i.e., $x_{i}(t)\to c_{\ell }(t)\;\forall \;v_{i}^{\ell }\in C_{\ell }$, given the appropriate initial conditions and after any transients have died out.

The symmetries in the graph fibration allow the system of ODEs admissible to the graph G to be reduced from a $\left|V_{G}\right|$ number of equations to a K number of equations for each fiber cluster. Equation (12) can be reduced to a system of equations for each of the fibers:

(13) $$\frac{dc_{i}}{dt}=F\left(c_{i},t\right)+\sum_{j\in V\left(c_{i}\right)}Q_{ij}H\left(c_{i},c_{j},t\right).$$

Here, Q is the adjacency matrix of the base of the graph obtained by the fibration.

The fibration formalism extends the automorphism symmetry groups of the graph.^5^ An automorphism is a permutation symmetry of the nodes of the graph that leaves invariant the adjacency of nodes. That is, the nodes permuted by the automorphism have the same in- and out-neighbors before and after the application of the automorphism. The analogous of fibers in fibrations are the orbits of the automorphisms. An orbit a node is the set of nodes obtained by the application of all the automorphisms of the graph.

Both orbits and fibers are balanced colorings of the graph. All orbits are fibers, but not all fibers are orbits. Thus, the fibers of the fibration capture more balanced colorings than the orbits of automorphisms. In short, fibration symmetries are rigorous extensions of automorphisms, and all automorphisms are fibration symmetries but the opposite is not always true.

This situation is exemplified in Fig. 4A. This graph has no automorphism. That is, there is no permutation of any nodes that leaves invariant the adjacency matrix. Therefore it has a trivial orbital (and balanced coloring) partition where each node is its own orbit or its own color. However, there is another minimal balanced coloring partition that is shown in the two colors in the center of Fig. 4A. This is the balanced coloring partition captured by the fibration, which is then applied to reduce the network to the base on the left of Fig. 4A.

A final fundamental difference between automorphism and fibration symmetry is that the former is a global symmetry of the network. That is, it is a permutation that constrains the global adjacency of nodes to be the same. However, the fibration is a local symmetry leaving invariant the input trees of the nodes, which are local views of the network of the nodes collapsed by the fibration. This fundamental property makes the fibration applicable to a larger set of complex networks than the more restricted automorphism.

The interplay between fibration symmetry and synchronization in networks via admissible ODEs provide profound insights into the dynamics of complex systems. The structural constraints imposed by fibration symmetry can dictate the synchronization patterns, where the quotation or base graph provides a simplified version of a larger networks leading to predictable and potentially controllable behavior in networks. This understanding has significant implications for the design and analysis of complex systems in various domains, ranging from biological networks (as in this paper) to engineered distributed systems ^5,6,11,12,15–17,57,59^

### 4.6 Symmetry Driven Repair Algorithm

In this section, we present a set of equations and inequalities based on the concepts discussed previously to construct our integer linear programming model for our problem, considering a specific graph, node clustering, parameters, and constraints. ^60,61^ We name this as the Symmetry-Driven Repair Algorithm or SymRep for short.

For this model, we consider a directed graph where we denote n=|V| and m=|E| as the number of nodes and directed edges, respectively. We also define

$$E^{C}=\{(i,j):i,j\in V,ij\notin E\}$$

as the set of node pairs between which no directed edge exists in G: these pairs indicate potential edges that could be added to the graph G. We define 𝒮 as a coloring of G, 𝒮 represents the sets dividing V, i.e., the various clusters of nodes.

The three types of decision variables in the model are:

$$\begin{array}{ll} r_{ij}\;\text{for}\;(i,j)\in E & \;\text{is}\;\text{a}\;\text{binary}\;\text{indicator}\;\text{for}\;\text{when}\;\text{edge}\;(i,j)\;\text{is}\;\text{removed;}\;\\ a_{ij}\;\text{for}\;(i,j)\in E^{C} & \;\text{is}\;\text{a}\;\text{binary}\;\text{indicator}\;\text{for}\;\text{when}\;\text{a}\;\text{new}\;\text{edge}\;(i,j)\;\text{is}\;\text{added;}\;\text{and}\;\\ s_{ijR}\;\text{for}\;i\in P,j\in Q,R,P,Q\in 𝒮 & \;\text{is}\;\text{a}\;\text{binary}\;\text{indicator}\;\text{that}\;i\;\text{and}\;j\;\text{has}\;\text{an}\;\text{imbalance}\;\text{in}\;\text{the}\;\text{color}\;\text{set}\;R. \end{array}$$

The objective function aims to minimize the weighted sum of edges removed and added, defined as

(14) $$f_{\alpha ,\beta }\left(r,a\right)=\alpha \sum_{ij\in E}r_{ij}+\beta \sum_{ij\in E^{C}}a_{ij}.$$

Constants α,β are parameters that adjust the importance between edge removal and addition in the objective. The main constraint ensures that 𝒮 represents a balanced coloring of the graph G, as defined by

(15) $$\begin{array}{l} \sum_{ip\in E:i\in S}\left(1-r_{ip}\right)+\sum_{ip\in E^{C}:i\in S}a_{ip}=\\ \;\sum_{iq\in E:i\in S}\left(1-r_{iq}\right)+\sum_{iq\in E^{C}:i\in S}a_{iq};\;p,q\in T;S,T\in 𝒮. \end{array}$$

This aims to ensure the nodes in the same division receive equal influence from all other divisions; this is the SymRep equivalent of Eq. (10). An optional constraint ensures that the in-degree of all nodes is at least one.


(16) $$\sum_{ip\in E}\left(1-r_{ip}\right)+\sum_{ip\in E^{C}}a_{ip}\geq 1,\;\;\;\;\;p\in V.$$

This helps avoid scenarios where nodes in a fiber are disconnected from the rest of the network. The following constraints are necessary but not sufficient for minimally balanced colorings Eq. (11).


(17) $$\begin{array}{r} \sum_{ip\in E:i\in R}\left(1-r_{ip}\right)+\sum_{ip\in E^{C}:i\in R}a_{ip}-\left(\sum_{iq\in E:i\in R}\left(1-r_{iq}\right)+\sum_{iq\in E^{C}:i\in R}a_{iq}\right)\geq s_{pqR}+ns_{qpR};\\ p\in S;\;q\in T;R,S,T\in 𝒮 \end{array}$$

(18) $$\begin{array}{r} \sum_{iq\in E:i\in R}\left(1-r_{iq}\right)+\sum_{iq\in E^{C}:i\in R}a_{iq}-\left(\sum_{ip\in E:i\in R}\left(1-r_{ip}\right)+\sum_{ip\in E^{C}:i\in R}a_{ip}\right)\geq s_{qpR}+ns_{pqR};\\ p\in S;q\in T;R,S,T\in 𝒮, \end{array}$$

(19) $$s_{pqR}+s_{qpR}\leq 1;\;\;p\in S;q\in T;R,S,T\in 𝒮,$$

(20a) $$\sum_{R\in 𝒮}\left(s_{pqR}+s_{qpR}\right)\geq 1;\;\;\;\;\;\;\;\;\;\;p\in S;q\in T;S,T\in 𝒮$$

(20b) $$\sum_{O\in 𝒮-\left(S\cup T\right)}\left(s_{pqO}+s_{qpO}\right)+\sum_{I\in \left(S\cup T\right)}\left|s_{pqI}-s_{qpI}\right|\geq 1;\;\;\;\;\;\;\;\;\;\;\;p\in S;q\in T;S,T\in 𝒮$$

These inequalities define the limits to ensure that two different divisions do not have the same influence, termed the unbalancing constraint. The entire model is defined as follows.

(21) $$min\left\{f_{\alpha ,\beta }\;:(15),(16),(17),(18),(19),(20),r_{ij},a_{k\ell },s_{pqR}\in \{0,1\},ij\in E,k\ell \in E^{C},p\in P,q\in Q,P\neq Q,R\in 𝒮\right\}.$$

where Eq. (20) within the equation above is a reference to only select one of its sub-equations. Eq. (20b) restricts feasible solutions and potentially better minimal balanced solutions relative to Eq. (20a) but is stronger with respect to the minimal balanced coloring property. In our implementation, we always try to find a solution to our networks by first implementing Eq. (20b) after which if it fails to produce a minimal colored solution, then Eq. (20a) is used. We solve the integer linear programs with the solver Gurobi. The implementation is available at https://github.com/makselab/PseudoBalancedColoring and https://osf.io/26u3g.

## Supplementary Material

Supplement 1

## Figures and Tables

**Table 1.** Solution metrics. The first two blocks in this table correspond to the best solutions found for the intersection between the community detection algorithm and the metric used to determine synchronization (Correlations vs *LoS*). The third block, located at the bottom corresponds to the first number of partitionings for the consensus method as visualized in Fig. 3F with an emphasis on showing the values before and after the most optimal partitioning found across all tested partitionings (cutoff at 0.55). The p-values indicate how often the partitioning performs equally or better than versions of the same partitioning with the labels of its nodes randomly shuffled. The values of *α* and *β* indicate the penalty values for which the algorithm found the optimal solution. The fiber column indicates the number of clusters for the given partitioning among motor neurons. These numbers do not include the three rigid clusters reserved for the inter-neurons.

Algorithm	Measure	Value	Modified	*α*	*β*	Fibers	P-value
Individual partitionings applied on Uncollapsed Varshney connectome							
Louvain	Corr.	Cov.	42.71%	1	1	2	0.023
Louvain	*LoS*	0.06	38.54%	1	1	3	0.007
Clique sync	Corr.	Cov.	40.62%	3	1	2	0.003
Clique sync	*LoS*	0.16	38.54%	1	1	3	0.008
Individual partitionings applied on Collapsed Varshney connectome							
Louvain	Corr.	Cov.	26.04%	1	2	2	0.019
Louvain	*LoS*	0.06	26.04%	1	2	3	0.033
Clique sync	Corr.	Cov.	25.00%	1	1	2	0.003
Clique sync	*LoS*	0.17	23.96%	1	1	3	0.007
Consensus partitionings applied on Collapsed Varshney connectome							
Consensus	Cutoff @	1.73	28.12%	1	2	3	0.157
Consensus	Cutoff @	0.85	28.12%	1	2	4	0.248
Consensus	Cutoff @	0.77	27.08%	1	2	5	0.211
Consensus	Cutoff @	0.58	27.08%	2	1	6	0.274
Consensus	Cutoff @	0.55	21.88%	1	1	7	0.033
Consensus	Cutoff @	0.50	23.96%	1	1	8	0.139
Consensus	Cutoff @	0.40	22.92%	1	1	9	0.143
Consensus	Cutoff @	0.34	20.83%	1	1	10	0.099

**Table 2.** Synchronization Measures in Time and Frequency Domains

	Time Domain	Frequency Domain
**With Directional Dependency**	Cross-correlation, Granger Causality, Transfer Entropy	Phase Locking Value, Directed Transfer Function, Partial Directed Coherence
**No Directional Dependency**	Pearson Correlation, Spearman’s Rank Correlation, Mutual Information	Coherence, Spectral Correlation, Phase Coherence

## Data Availability

All data and code are available at https://github.com/MakseLab, https://osf.io and http://kcorebrain.com. In particular, an implementation of the algorithm to find minimal balanced colorings in a graph in the form of an R package is available at https://github.com/makselab/fibrationSymmetries and https://osf.io/z793h/. An implementation of the MILP solver is available at https://github.com/makselab/PseudoBalancedColoring and https://osf.io/26u3g.

## Significance Statement

Complex biological networks exhibit an intricate relationship between the structural composition of their elements’ interaction and the whole system’s functionality. This is particularly true in neuroscience, where the multiscale organization of neurons gives place to a multitude of hierarchical functionalities responsible for the nervous system interacting with the environment in which it is embedded. In this study, we focus on the chemical synaptic network of the backwards-crawling locomotion circuitry of the worm C. elegans to correlate structural clusters of neurons with dynamic patterns of their synchronization. Our findings reveal functional building blocks in the animal’s motor periphery, providing new testable hypotheses on how descending interneuron circuits communicate with the motor periphery to control behavior.
