# Supplementary material for: Symmetries and synchronization from whole-neural activity in C. elegans connectome: Integration of functional and structural networks
Source: ArXiv. 2024 Sep 4:arXiv:2409.02682v1. Preprint. [Version 1] (PMC11398546)
Supplement: Supplement 1 [file NIHPP2409.02682v1-supplement-1.pdf]

## Supporting information

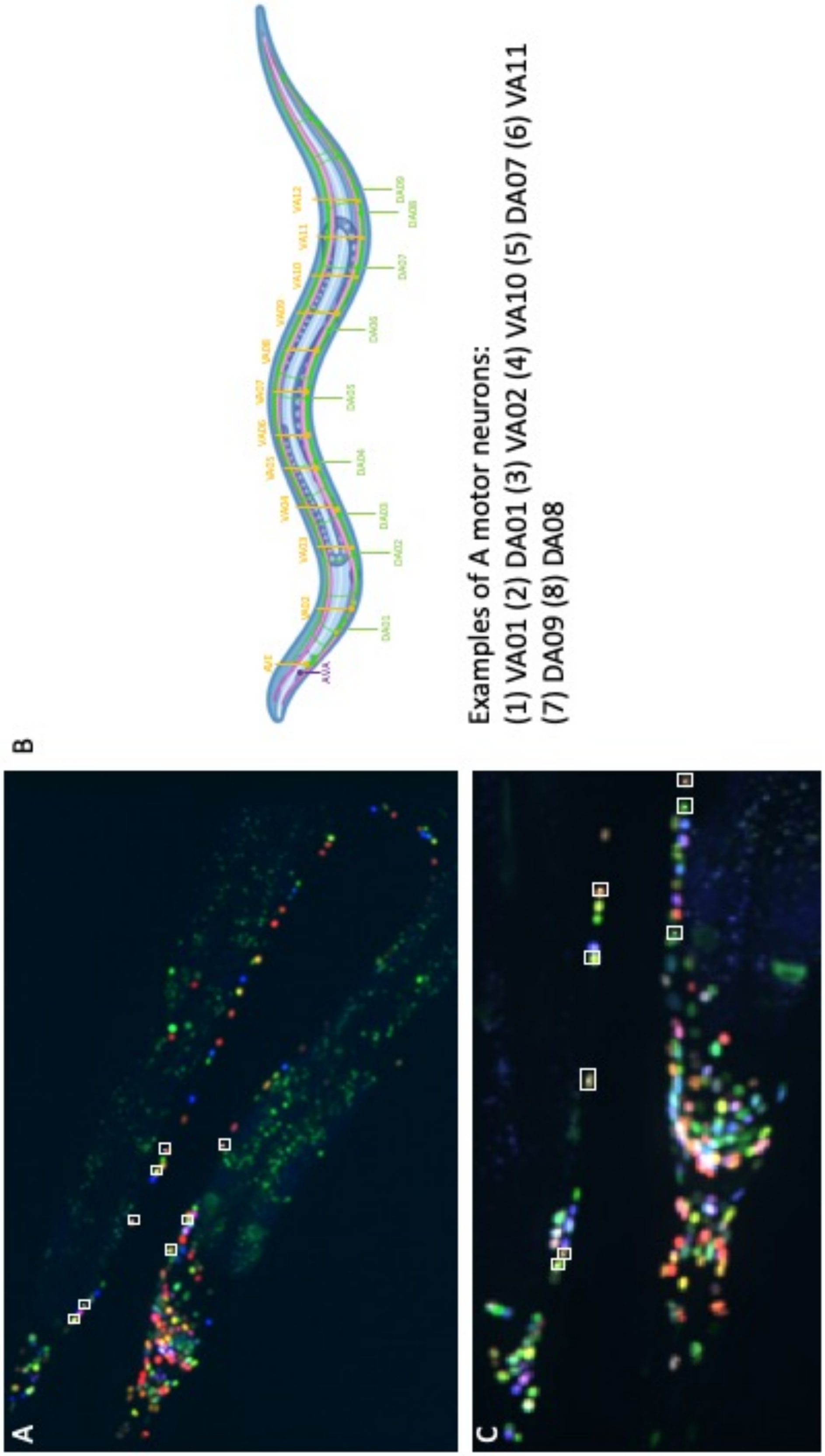


**Fig 7. Motor neuron examples through NeuroPAL labeling.** (A) 20x magnification high-quality NeuroPAL Z stack labeling with some specific examples of reverse motor neurons are also present in (C). (B) Schematic representing the anatomical location of all motor neurons of interest (DAs and VAs). (C) 40x magnification high-quality NeuroPAL Z stacks labeling reverse motor neurons also present in (A).
